# Composition and Biochemical Determinants of Renal Stones: A Comprehensive Analysis of Urinary and Serum Mineral Profiles

**DOI:** 10.5152/tud.2026.26015

**Published:** 2026-05-20

**Authors:** Pranav Prakash, Shikhar Agarwal, Rajeev Sarpal, Ashok Kumar Dogra, Archana Prakash

**Affiliations:** 1 Department of General Surgery, Himalayan Institute of Medical Sciences, Swami Rama Himalayan University, Dehradun, Uttarakhand, India; 2 Department of Urology, Himalayan Institute of Medical Sciences, Swami Rama Himalayan University, Dehradun, Uttarakhand, India; 3 Department of Biochemistry, Government Medical College, Srinagar, Jammu and Kashmir, UT; 4 Department of Biochemistry, Himalayan Institute of Medical Sciences, Swami Rama Himalayan University, Dehradun, Uttarakhand, India

**Keywords:** Renal stones, stone composition, biochemical parameters, calcium oxalate

## Abstract

**Objective:**

Renal stone formation is closely linked to disturbances in urinary and serum mineral metabolism. Analysis of stone composition along with biochemical parameters is essential for understanding the pathogenesis of different stone types.

**Methods:**

A prospective observational study was conducted among 371 patients with renal stones. Stone composition was analyzed using Fourier transform infrared spectroscopy, X-ray diffraction, and scanning electron microscopy- Energy-Dispersive X-ray Spectroscopy techniques. Serum and 24-hour urinary biochemical parameters, including calcium, oxalate, citrate, phosphate, uric acid, pH, and urine volume, were evaluated. Statistical analysis was performed to assess differences and correlations between stone types and biochemical variables.

**Results:**

The mean age was 45.3 years, with male predominance and 39.9% recurrence. Calcium oxalate monohydrate was most common. Stone types differed significantly in urinary volume, pH, and mineral excretion. Uric acid stones showed low urine pH and volume, while calcium phosphate stones had higher calcium excretion and alkaline pH, with related serum differences.

**Conclusion:**

Renal stone composition is strongly associated with specific urinary and serum biochemical abnormalities. Metabolic evaluation is crucial for identifying etiological factors and for guiding individualized preventive strategies in stone disease management.

**Main Points**

- Stone formation is linked to type-specific metabolic disturbances influenced by demographics, diet, and environment.
- Recognizing these biochemical patterns is crucial for understanding why stones form in each patient.
- Full metabolic testing helps identify underlying causes, guides personalized prevention strategies, reduces recurrence, and improves long-term outcomes.

## Introduction

Renal stone disease is a common urological condition worldwide and represents a growing public health concern due to its high prevalence, recurrence, and associated morbidity. Epidemiological studies have shown a steady rise in the incidence of nephrolithiasis across different populations, attributed to changes in diet, lifestyle, and metabolic health.^1^^-^^3^ Beyond acute symptoms, kidney stone disease has also been linked to long-term complications, including chronic kidney disease, emphasizing the importance of understanding its underlying mechanisms.^4^^,^^5^

The pathogenesis of renal stone formation is complex and involves a sequence of physicochemical events, including urinary supersaturation, crystal nucleation, growth, aggregation, and retention within the renal tubules.^6^^,^^7^ These processes are strongly influenced by metabolic and biochemical abnormalities such as hypercalciuria, hyperoxaluria, hypocitraturia, altered urinary pH, and abnormalities in uric acid metabolism.^8^^,^^9^ Nephrolithiasis is increasingly recognized as a systemic metabolic disorder rather than a localized renal condition, with stone composition reflecting the dominant biochemical imbalance present during stone formation.^9^^,^^5^

Analysis of renal stone composition provides critical insight into the etiology of stone disease and plays a central role in guiding preventive strategies. Calcium oxalate stones constitute the majority of renal calculi, followed by calcium phosphate and uric acid stones, each associated with distinct urinary and serum biochemical profiles.^10^ Variations in urinary pH, mineral excretion, and metabolic risk factors have been shown to influence stone type and morphology, underscoring the importance of detailed compositional and metabolic evaluation.^6^^,^^11^ However, data correlating detailed stone composition with comprehensive metabolic parameters within a single cohort remain limited.^12^ The present study therefore aims to evaluate the association between renal stone composition and urinary and serum biochemical abnormalities.

**Objectives:** To analyze renal stone composition and its relationship with urinary and serum biochemical parameters, examining the distribution of stone types and associated metabolic abnormalities.

**Expected Outcomes:** The study aimed to identify predominant stone types, define stone-specific biochemical patterns, and link metabolic abnormalities to stone composition, enhancing understanding of nephrolithiasis and supporting biochemical evaluation for prevention and management.

## Material and Methods

**Study Design: **A prospective cross-sectional study with correlational analysis was conducted in the Department of General Surgery/Urology, Himalayan Institute of Medical Sciences, Swami Ram Nagar **Dehradun, **Uttarakhand, India, from November 2022 to October 2023 (24 months).

**Statement** **of Ethics: **The study was approved by the Institutional Ethics Committee of Himalayan Institute of Medical Sciences, Swami Ram Nagar, Dehradun, Uttarakhand, India ( SRHU/HIMS/Ethics/2022/335) and conducted over 24 months (November 2021–October 2023) in accordance with the Declaration of Helsinki and Good Clinical Practice guidelines. Written informed consent was obtained from all participants, who were informed of their right to withdraw without affecting clinical care. Patient data were de-identified and securely stored, with restricted access to authorized personnel only.

**Sample Size Calculation:** The sample size (n) was calculated using the formula for estimating a population proportion with specified precision: **n = *Z*²₁₋*ₐ*/₂ × *p* × (1 − *p*) / *d*²**

Where *Z*₁₋*ₐ*/₂ = standard normal variate (at 5% type I error, *Z* = 1.96), *p* = prevalence, *d* = precision

Based on previous studies, the expected prevalence of calcium oxalate stones is approximately 70% (24). Using a precision of 5% and a confidence level of 95%:

n = (1.96)² × 0.70 × (1−0.70) / (0.05)² = 322.69

Accounting for 15% dropouts, the final target was 371 patients.

For the correlational analysis: **n = [(*Zₐ* + *Z*₁₋*ᵦ*) / *C*]² + 3**

Where *Zₐ* = 1.96 at *α* = 0.05, *Z*₁₋*ᵦ* = 0.84 at *β* = 0.20, power = 80%, *C* = 0.5 × ln [(1 + *r*)/(1 − *r*)], r = 0.3 (Based on previous studies, a moderate correlation between urinary calcium and calcium oxalate stone burden is anticipated):

*C* = 0.5 × ln [(1 + 0.3) / (1−0.3)] = 0.5 × ln [1.857] = 0.5 × 0.619 = 0.310

n = [(1.96 + 0.84) / 0.310]² + 3 = 87.58 ≈ 88

Therefore, a minimum of 88 patients with calcium oxalate stones was required for correlation analysis. Since the overall sample size exceeded this number and approximately 70% of stones were expected to be calcium oxalate, the total sample of 371 was sufficient.

**Study Population: **Adults aged 18-75 years with confirmed renal stones who underwent stone removal and could provide consent for complete metabolic evaluation were included. Patients with active urinary tract infection, advanced renal failure (eGFR <30 mL/min/1.73 m²), pregnancy/lactation, malignancy, urinary tract abnormalities, difficulty with 24-hour urine collection, or recent use of medications affecting urinary biochemistry (thiazides, allopurinol, potassium citrate, and calcium) were excluded.

**Recruitment Procedure: **Eligible patients scheduled for stone removal were consecutively recruited after written informed consent. A standardized recruitment protocol was followed to minimize selection bias.

### Data Collection

**Clinical and Demographic Data: **Information on age, sex, BMI, medical history, stone recurrence, dietary habits, lifestyle factors, and clinical stone characteristics (size, site, and number) was collected using a structured case record form.

**Stone Analysis: **Retrieved stones were analyzed using Fourier transform infrared spectroscopy for composition, X-ray diffraction (XRD) for crystalline structure, and scanning electron microscopy with EDXA (selected samples) for morphology and elemental composition. Stones were classified by predominant component (>50%) as calcium oxalate monohydrate, calcium oxalate dihydrate, calcium phosphate, uric acid, struvite, cystine, or mixed stones.

**Biochemical Evaluation: **Biochemical assessments were performed 4-6 weeks post-stone removal to allow metabolic stabilization and to avoid transient changes following stone removal procedures. Serum parameters included calcium, phosphorus, magnesium, uric acid, creatinine, electrolytes, parathyroid hormone, vitamin D, alkaline phosphatase, albumin, bicarbonate, and complete blood count, which was included to assess infection and inflammatory status that may influence stone formation and patient condition. Two 24-hour urine collections were analyzed for volume, pH, calcium, oxalate, citrate, phosphate, magnesium, uric acid, electrolytes, ammonium, sulfate, creatinine, protein, and cystine (selected cases), along with spot urine ratios. Urinary supersaturation indices for calcium oxalate, calcium phosphate, and uric acid were calculated using the Equil2 program.

**Quality Control Measures: **All analyses were performed in duplicate with regular equipment calibration. Laboratory personnel were blinded to clinical data, and internal and inter-laboratory quality controls were implemented.

**Data Management and Statistical Analysis:** Data were securely stored with double data entry and validation checks. Statistical analyses were performed using IBM SPSS Statistics for Windows, Version 22.0 (IBM SPSS Corp.; Armonk, NY, USA).

**Biological Sample Storage and Future Research: **Participants were asked for separate consent for the storage of residual stone samples and biological specimens for potential future research. If consent was provided, samples were stored in a secure biorepository for up to 10 years.

## Results

Table 1 presents baseline characteristics of renal stone patients. The mean age was 45.3 ± 14.2 years, with male predominance (59.8%) and 39.9% having recurrent stones. Most patients were overweight/obese (65%, mean BMI 27.4 ± 5.1 kg/m²). Dysuria was common (70.1%), and multiple stones occurred in 39.9%. The kidney was the most frequent site (55%), mainly right-sided (45%). Calcium oxalate monohydrate was the most common stone type (49.9%), followed by calcium oxalate dihydrate (19.9%) and calcium phosphate (10%).

Table 2 compares the urinary mineral profile across different renal stone types. Significant differences were observed in 24-hour urine volume (*P* = .032), highest in calcium phosphate stone formers (1890 ± 690 mL) and lowest in uric acid stone formers (1420 ± 510 mL). Urinary pH varied significantly, lowest in uric acid stones (5.3 ± 0.4) and highest in struvite stones (7.2 ± 0.8). Calcium excretion differed significantly (*P* < .001), highest in calcium phosphate (338 ± 116 mg/24 hours) and lowest in uric acid stones (186 ± 74 mg/24 hours). Oxalate, citrate, and phosphate levels also showed significant variation, while magnesium, sodium, and potassium did not (*P* > .05).

Table 3 compares serum mineral parameters across stone types. Uric acid was highest in uric acid stone formers (7.2 ± 1.8 mg/dL), while vitamin D levels varied significantly, highest in calcium phosphate stones (32 ± 16 ng/mL) and lowest in uric acid stones (24 ± 8 ng/mL). No significant differences were observed for serum calcium, phosphorus, magnesium, Parathyroid Hormone (PTH), or albumin.

Table 4 shows significant variation in urinary abnormalities across stone types. Hypercalciuria was highest in calcium phosphate stones (70.3%) and lowest in uric acid stones (15.2%) (*P* < .001). Hyperoxaluria was most common in mixed calcium oxalate stones (57.7%) and least in uric acid stones (12.1%) (*P* < .001). Hypocitraturia was highest in mixed calcium oxalate stones (61.5%, *P* = .024). Hyperuricosuria and low urine volume were most frequent in uric acid stones (84.8% and 54.5%, *P* < .001 and *P* = .003, respectively). Abnormal urinary pH was prevalent in struvite (81.8%) and uric acid stones (66.7%, *P* < .001).

Table 5 shows significant correlations between stone types and urinary parameters. Calcium oxalate monohydrate stones correlated positively with urinary oxalate (*r* = 0.52) and negatively with pH (*r* = −0.38), while calcium oxalate dihydrate stones correlated positively with calcium (*r* = 0.48) and negatively with citrate (*r* = −0.32). Calcium phosphate stones were associated with higher pH (*r* = 0.61) and lower oxalate (*r* = −0.41). Uric acid stones correlated with higher urinary uric acid (*r* = 0.64) and lower pH (*r* = −0.58). Struvite stones were linked to high pH (*r* = 0.72) and lower calcium (*r* = −0.36).

## Discussion

This study comprehensively assessed the relationship between renal stone composition and biochemical parameters in a diverse stone-forming population. Significant differences in urinary volume, pH, mineral excretion, and selected serum markers were observed across stone types, reflecting the metabolic heterogeneity of nephrolithiasis and highlighting the importance of thorough biochemical evaluation for prevention and management.

Renal stones were most common in middle-aged adults, with male predominance and high rates of overweight/obesity, supporting previous studies by Scales et al., Romero et al., and Soucie et al.^1^^,^^2^^,^^13^ The recurrence rate of ~40% reflects the chronic nature of nephrolithiasis, as noted by Trinchieri et al.^14^ Calcium oxalate monohydrate stones predominated, followed by dihydrate forms, while uric acid and struvite stones fell within ranges reported by Coe et al. and Worcester et al., Daudon et al., and Sorokin et al.^7^^,^^9^^,^^3^ A summer peak was observed, likely due to dehydration, consistent with Boscolo-Berto et al. and Eisner et al.^15^^,^^16^ Rare metabolic disorders such as xanthine oxidase deficiency have also been reported to cause urinary stone formation due to abnormalities in purine metabolism, further supporting the role of biochemical disturbances in stone etiology.^17^

Urinary biochemical patterns varied by stone type. Calcium phosphate stones were associated with higher urinary calcium and alkaline pH, while uric acid stones showed lower pH and volume, consistent with Tiselius et al., Pak et al., and Bargagli et al.^6^^,^^8^^,^^18^ Hypercalciuria, hyperoxaluria, hypocitraturia, and hyperuricosuria distributions mirrored patterns reported by Ferraro et al. and Moreira et al.^19^^,^^20^ Serum uric acid was elevated in uric acid stone formers, and vitamin D levels varied across subtypes, as noted by Abate et al. and Ticinesi et al.^21^^,^^22^ Correlation analyses confirmed stone-specific metabolic associations, consistent with Zhang et al.^23^ These findings reinforce the need for comprehensive metabolic evaluation to guide individualized prevention and reduce recurrence risk. In addition, previous studies have highlighted the importance of appropriate management strategies, including conservative approaches in selected patients, emphasizing the need for careful patient selection and long-term monitoring in stone disease.^24^

### Limitations, Strengths, and Future Directions

This study has several limitations that should be acknowledged. Its cross-sectional design captures metabolic parameters at a single time point and may not reflect the temporal variability of stone formation. The single-center setting, potential selection bias, reliance on self-reported dietary information, variability in stone composition analysis, incomplete urine collections, and the absence of assessment of certain urinary inhibitors and promoters may limit generalizability and introduce recall or measurement bias. To address these issues, standardized recruitment procedures, validated dietary assessment tools, strict laboratory quality control, urine completeness verification, and detailed patient documentation were employed. Despite these constraints, the study is strengthened by a large and diverse cohort (n = 371), inclusion of multiple stone types, and comprehensive biochemical profiling, allowing robust evaluation of metabolic patterns associated with nephrolithiasis. Future research should adopt multi-center, longitudinal designs, incorporate detailed dietary and environmental factors, and include additional urinary biomarkers to enhance predictive models and support personalized stone prevention strategies.

This study highlights the strong link between renal stone composition and distinct urinary and serum biochemical profiles. Calcium-based and uric acid stones show characteristic metabolic abnormalities, such as variations in urinary pH, mineral excretion, and serum markers, emphasizing that stone formation is driven by type-specific metabolic disturbances influenced by demographic, dietary, and environmental factors.

Understanding these patterns is essential for effective management of nephrolithiasis. Comprehensive metabolic evaluation helps identify underlying causes, guide personalized preventive strategies, and reduce recurrence, ultimately improving long-term patient outcomes.

## Figures and Tables

**Table 1.** Baseline Demographic, Clinical Characteristics, and Renal Stone Composition of the Study Population (n = 371)

**Parameter**		**Number of Patients (n)**	**Percentage (%)**
Age (years)	Mean ± SD	45.3 ± 14.2 years	
	18-40	130	35
	41-60	167	45
	≥60	74	20
Gender	Male	222	59.80
	Female	149	40.20
Recurrence	First episode	223	60.10
	Recurrent	148	39.90
BMI	Mean ± SD	27.4 ± 5.1 kg/m²	
	Normal	130	35
	Overweight	160	43.10
	Obese	81	21.90
Dysuria	Present	260	70.10
	Absent	111	29.90
Stone multiplicity	Single	223	60.10
	Multiple	148	39.90
Site of upper tract stones	Kidney	204	55
	Ureter	130	35
	Both	37	10
Side of upper tract obstruction	Right	167	45
	Left	156	42
	Bilateral	48	13
Large stones in bulk	Present	93	25.10
	Absent	278	74.90
Stone composition	Calcium oxalate monohydrate	185	49.90
	Other types	186	50.10
Urinary tract infection history	Present	89	24
	Absent	282	76
Seasonal distribution	Summer	150	40.40
	Winter	74	19.90
	Spring	82	22.10
	Fall	65	17.60
Stone type	Calcium oxalate monohydrate	185	49.9
	Calcium oxalate dihydrate	74	19.9
	Mixed calcium oxalate	26	7
	Calcium phosphate	37	10
	Uric acid	33	8.9
	Struvite	11	3
	Cystine	4	1.1
	Mixed/other	1	0.3

**Table 2.** Urinary Mineral Profile by Stone Type (Mean ± SD)

**Parameter**	**CaOx Mono (n = 185)**	**CaOx Di (n = 74)**	**Mixed CaOx (n = 26)**	**CaP (n = 37)**	**Uric Acid (n = 33)**	**Struvite (n = 11)**	P^*^
Volume (mL/24 hours)	1650 ± 620	1720 ± 580	1580 ± 540	1890 ± 690	1420 ± 510	1760 ± 630	0.032
pH	5.8 ± 0.4	6.1 ± 0.5	5.9 ± 0.6	6.7 ± 0.6	5.3 ± 0.4	7.2 ± 0.8	<.001
Ca (mg/24 hours)	284 ± 92	312 ± 104	298 ± 88	338 ± 116	186 ± 74	205 ± 82	<.001
Oxalate (mg/24 hours)	43 ± 15	38 ± 14	46 ± 18	31 ± 10	29 ± 12	32 ± 14	<.001
Citrate (mg/24 hours)	450 ± 210	520 ± 240	390 ± 180	480 ± 230	420 ± 190	510 ± 250	.017
Phosphate (mg/24 hours)	890 ± 320	910 ± 290	870 ± 310	980 ± 340	830 ± 270	940 ± 310	.043
Magnesium (mg/24 hours)	88 ± 32	96 ± 38	84 ± 30	92 ± 36	78 ± 28	90 ± 34	.082
Uric Acid (mg/24 hours)	620 ± 180	590 ± 160	650 ± 190	580 ± 150	780 ± 210	540 ± 140	<.001
Sodium (mEq/24 hours)	168 ± 58	182 ± 62	176 ± 64	164 ± 56	186 ± 66	172 ± 60	.097
Potassium (mEq/24 hours)	58 ± 22	62 ± 24	54 ± 20	64 ± 26	52 ± 18	60 ± 22	.115

*Signifies significant *P* value< .05. CaP, Calcium phosphate.

**Table 3.** Serum Mineral Parameters by Stone Type (Mean ± SD)

**Parameter**	**CaOx Mono (n = 185)**	**CaOx Di (n = 74)**	**Mixed CaOx (n = 26)**	**CaP (n = 37)**	**Uric Acid (n = 33)**	**Struvite (n = 11)**	P^*^
Ca (mg/dL)	9.4 ± 0.5	9.5 ± 0.6	9.3 ± 0.4	9.6 ± 0.7	9.2 ± 0.4	9.1 ± 0.5	.062
Phosphorus (mg/dL)	3.4 ± 0.6	3.3 ± 0.5	3.5 ± 0.7	3.2 ± 0.4	3.6 ± 0.8	3.4 ± 0.6	.086
Magnesium (mg/dL)	2.1 ± 0.3	2.2 ± 0.4	2.0 ± 0.3	2.1 ± 0.4	2.0 ± 0.3	2.1 ± 0.4	.243
Uric acid (mg/dL)	5.8 ± 1.4	5.6 ± 1.3	6.0 ± 1.5	5.4 ± 1.2	7.2 ± 1.8	5.5 ± 1.2	<.01
PTH (pg/mL)	42 ± 18	40 ± 16	44 ± 20	38 ± 14	44 ± 20	46 ± 22	.167
Vitamin D (mg/mL)	28 ± 12	30 ± 14	26 ± 10	32 ± 16	24 ± 8	26 ± 10	.018
Albumin (g/dL)	4.2 ± 0.4	4.3 ± 0.5	4.1 ± 0.3	4.3 ± 0.5	4.0 ± 0.3	4.1 ± 0.4	.089

*Signifies significant *P* value < .05.

**Table 4.** Prevalence of Urinary Biochemical Abnormalities by Stone Type

**Abnormality**	**CaOx Mono (n = 185) (%)**	**CaOx Di (n = 74) (%)**	**Mixed CaOx (n = 26) **(%)****	**CaP (n = 37) **(%)****	**Uric Acid (n = 33) **(%)****	**Struvite (n = 11) **(%)****	***P***
Hypercalciuria	98 (53.0)	48 (64.9)	14 (53.8)	26 (70.3)	5 (15.2)	3 (27.3)	<.001*
Hyperoxaluria	76 (41.1)	22 (29.7)	15 (57.7)	6 (16.2)	4 (12.1)	2 (18.2)	<.001*
Hypocitraturia	82 (44.3)	24 (32.4)	16 (61.5)	14 (37.8)	15 (45.5)	3 (27.3)	.024*
Hyperuricosuria	64 (34.6)	20 (27.0)	12 (46.2)	8 (21.6)	28 (84.8)	2 (18.2)	<.001*
Low urine volume	58 (31.4)	18 (24.3)	10 (38.5)	6 (16.2)	18 (54.5)	2 (18.2)	.003*
Abnormal pH	42 (22.7)	12 (16.2)	8 (30.8)	24 (64.9)	22 (66.7)	9 (81.8)	<.001*

*Signifies significant *P* value < .05.

**Table 5.** Correlation Between Stone Composition and Urinary Parameters

**Stone Type**	**Strongest Positive Correlations**	***r***	P^*^	**Strongest Negative Correlations**	***r***	P^*^
CaOx monohydrate	Urinary oxalate	0.52	<.001	Urinary pH	−0.38	<.001
CaOx dihydrate	Urinary calcium	0.48	<.001	Urinary citrate	−0.32	.002
Calcium phosphate	Urinary pH	0.61	<.001	Urinary oxalate	−0.41	<.001
Uric acid	Urinary uric acid	0.64	<.001	Urinary pH	−0.58	<.001
Struvite	Urinary pH	0.72	<.001	Urinary calcium	−0.36	.004

*Signifies significant *P* value < .05.

## Data Availability

The data that support the findings of this study are available on request from the corresponding author.
